# Comment on Gao, W., *et al*. “Efficient One-Pot Synthesis of 5-Chloromethylfurfural (CMF) from Carbohydrates in Mild Biphasic Systems”, *Molecules* 2013, *18*, 7675-7685

**DOI:** 10.3390/molecules19011367

**Published:** 2014-01-22

**Authors:** Mark Mascal

**Affiliations:** Department of Chemistry, University of California Davis, 1 Shields Avenue, Davis, CA 95616, USA; E-Mail: mjmascal@ucdavis.edu; Tel.: +1-530-754-5373

In a recent paper entitled “Efficient One-Pot Synthesis of 5-Chloromethylfurfural (CMF) from Carbohydrates in Mild Biphasic Systems,” published in *Molecules* [1], Gao and coworkers describe the use of a biphasic aq. HCl-H_3_PO_4_/CHCl_3_ reagent for the preparation of CMF from various feedstocks. The maximum yield (46.8%) was obtained from fructose by reaction at 45 °C for 20 h. While sucrose gave a similar yield, the same reaction with glucose and cellulose gave 7.3% and 7.8% yields, respectively. Remarkably, the same process applied to Kraft pulp and powdered wood samples gave between 16.0% and 31.4% CMF, based on sugar content. Looking to the Experimental section for insight into this unusual outcome, the statement, “*the procedure of treating lignocellulose sample (Table 6) was almost the same as the carbohydrate, except adding the selected simple 1.0 mg each trial* ” *[sic]* appears, which is difficult to interpret.

Given the above context, the publication of the present Letter is concerned mainly with the Introduction to the paper, which first mentions the conversion of 5-(hydroxymethyl)furfural (HMF) into CMF by the action of dry hydrogen halides based on work in reference [13], and then states that “*while the conversion of cellulose into CMF was low (12%), a substantially higher yield (48%) was obtained for the preparation of BMF when dry HBr was employed,*” further citing references [14–16]. Contrary to what is stated above, however, reference [13] (Sanda *et al*., *Carbohydr. Res.* **1989**, *187*, 15–23) describes the production of CMF from HMF in up to 87% yield, whereas reference [14] (Sanda *et al*., *Synthesis* **1992**, *6*, 541–542) involves the reaction of HMF with POCl_3_, with conversion to CMF in up to 92% yield. Reference [15] (Canas *et al*., *J. Sep. Sci.* **2003**, *26*, 496–502) contains no mention of any halogenated furfural whatsoever. Reference [16] (Hibbert *et al*., *J. Am. Chem. Soc.* **1923**, *45*, 176–182) reports a 56% yield of 5-(bromomethyl)furfural (BMF) from cellulose and dry HBr. Cited later, reference [17] (Kumari *et al*., *Eur. J. Org. Chem.* **2011**, *7*, 1266–1270) reports 82% and 80% yields of BMF from fructose and cellulose, respectively, in a biphasic reactor, and finally reference [18] (Brasholz, *et al*., *Green Chem.* **2011**, *13*, 1114–1117) reports yields of CMF up to 81% from fructose in a flow reactor, again in a biphasic system.

Our main concerns however stem from the authors’ citation, or more precisely lack of citation, of our most relevant work in this field. The authors state the following: “*Considering the importance of these compounds, Mascal et al. recently reported the synthesis of CMF from cellulose treated by HCl-LiCl and successive continuous extraction [2]. Unfortunately, 5-(chloromethyl)furfural, 2-(2-hydroxyacetyl)furan, 5-(hydroxylmethyl), furfural and levulinic acid were also produced with this system.*” The citation (paper reference [2]), is to Mascal and Nikitin, *Angew. Chem. Int. Ed.* **2008**, *47*, 7924–7926. This paper describes the production of CMF in 71%–76% yield in a biphasic HCl/ClCH_2_CH_2_Cl reactor including 5% LiCl in the aqueous phase, and employing continuous solvent extraction. Depending on the feedstock (glucose, sucrose or cellulose), an *additional* 14%–18% yield of a mixture of HMF, 2-(hydroxyacetyl)furan, and levulinic acid was in fact observed. However, in follow-up work (Mascal and Nikitin, *ChemSusChem* **2009**, *2*, 859–861, [2] in this Letter), we reported a substantially improved process, yielding 80%–90% CMF alongside 5%–8% levulinc acid from glucose, sucrose, cellulose or corn stover feedstocks. No LiCl was used, and no HMF or hydroxyacetylfuran was observed. No continuous extraction was required, and the reaction was complete within 1–3 h. The CMF could be isolated in a pure state by simply evaporating the solvent, and the small amount of levulinic acid by-product could be isolated by extraction of the aqueous phase, if desired. This work was not cited.

The authors continue their introduction with these words: “*Despite the numerous efforts aimed at these transformations, each of them suffers from at least one of the following limitations: diverse by-products in significant yields that reduce the selectivity of the reaction and its economics, low conversions and yields, harsh reaction conditions (dry hydrogen halide, relative high temperature), requirements for large amounts of costly reagents (LiCl, LiBr), prolonged reaction times and tedious operations with complex set ups (continuous extraction). These drawbacks seriously hamper their potential industrial applications.*” We take issue with each of these statements as follows:

(1) “*Diverse by-products in significant yields that reduce the selectivity of the reaction and its economics*.” Our initial report did include the description of by-products, but considering that the CMF yield even in this case was between 71%–76%, the presence of these by-products does not impact the economics of the process as much as would a poor yield of CMF in the first place (say <50%). In our follow-up work [2] the CMF yield was increased to between 80%–90% (depending on the feedstock), and only a small quantity of levulinic acid by-product (which, by the way, is considered a valuable platform chemical in its own right) was observed.
(2) “*Low conversions and yields.*” It cannot escape notice that the actual reported yields of halomethylfurfurals in the papers cited in the Introduction to [1] (56%–92% yield) are all higher than that described in the paper [1] itself.
(3) “*Harsh reaction conditions (dry hydrogen halide, relative high temperature).*” The use of dry hydrogen halides is actually easier to accommodate industrially than aqueous HX acids in terms of reactor materials required. The use of hydrochloric acid, either wet or dry, is necessary for this process in any case, and it has been in common practice in the chemical industry for many years. Regarding temperature, in none of the cited papers is the reaction carried out above 100 °C.
(4) “*Requirements for large amounts of costly reagents (LiCl, LiBr).*” In our original work (paper reference [2]), we used 5% LiCl, whereas Kumari and coworkers in reference [17] appear to use 6.7% LiBr. These are not large amounts, nor are these salts particularly costly (current bulk prices range between US$ 0.09–0.15 per gram). As noted, in our follow-up work [2], no salt is used at all.
(5) “*Prolonged reaction times and tedious operations with complex set ups (continuous extraction).*” Here, it can be said that our original work (paper reference [2]) did indeed involve prolonged reaction times, up to 30 h. We would however suggest that continuous extraction does not involve a particularly tedious operation or complex set up; in fact, in our laboratory, it involves a single piece of glassware (the extractor) and two round-bottomed flasks. Finally, again, this was done away with in the subsequent work, and the reaction time was reduced to no more than 3 h [2].
(6) “*These drawbacks seriously hamper their potential industrial applications.*” The authors may be pleased to learn that the CMF process described in [2] and the corresponding patent [3] has now been piloted, and a multi-ton production facility is planned to begin operation in 2014.

To conclude, while the above discussed paper of Gao, *et al*. [1] describes a useful study in its own right, it fails to cite relevant prior art, *i.e.*, our work [2]. Further, Gao, *et al*. [1] also fail to correctly represent the prior art they do cite. Outcomes in references [13–18] are not described accurately, and of the six criticisms the authors dispense in their Introduction, only (1) and (5) have any credibility, and even then only in part. Had paper [2] been taken into account, *none* of these critiques would have been valid.
